# Tranexamic acid reduces blood cost in long-segment spinal fusion surgery: A randomized controlled study protocol: Partial Retraction

**DOI:** 10.1097/MD.0000000000024983

**Published:** 2021-02-26

In the article, “Tranexamic acid reduces blood cost in long-segment spinal fusion surgery: A randomized controlled study protocol”,^[1]^ which appears in Volume 99, Issue 37 of *Medicine*, Table 1 was not provided and the article inadvertently published missing this content. The author has not responded to multiple attempts to secure the missing table, therefore it is being retracted from the article.
